# Curcumin Mimics the Neurocognitive and Anti-Inflammatory Effects of Caloric Restriction in a Mouse Model of Midlife Obesity

**DOI:** 10.1371/journal.pone.0140431

**Published:** 2015-10-16

**Authors:** Marjana Rahman Sarker, Susan Franks, Nathalie Sumien, Nopporn Thangthaeng, Frank Filipetto, Michael Forster

**Affiliations:** 1 Department of Pharmacology and Neuroscience, Institute for Aging and Alzheimer’s Disease Research (IAADR), University of North Texas Health Science Center, Fort Worth, Texas, United States of America; 2 Family Medicine, Texas College of Osteopathic Medicine, University of North Texas Health Science Center, Fort Worth, Texas, United States of America; Consiglio Nazionale delle Ricerche, ITALY

## Abstract

Dietary curcumin was studied for its potential to decrease adiposity and reverse obesity- associated cognitive impairment in a mouse model of midlife sedentary obesity. We hypothesized that curcumin intake, by decreasing adiposity, would improve cognitive function in a manner comparable to caloric restriction (CR), a weight loss regimen. 15-month-old male C57BL/6 mice were assigned in groups to receive the following dietary regimens for 12 weeks: (i) a base diet (Ain93M) fed ad libitum (AL), (ii) the base diet restricted to 70% of ad libitum (CR) or (iii) the base diet containing curcumin fed AL (1000 mg/kg diet, CURAL). Blood markers of inflammation, interleukin 6 (IL-6) and C-reactive protein (CRP), as well as an indicator of redox stress (GSH: GSSG ratio), were determined at different time points during the treatments, and visceral and subcutaneous adipose tissue were measured upon completion of the experiment. After 8 weeks of dietary treatment, the mice were tested for spatial cognition (Morris water maze) and cognitive flexibility (discriminated active avoidance). The CR group showed significant weight loss and reduced adiposity, whereas CURAL mice had stable weight throughout the experiment, consumed more food than the AL group, with no reduction of adiposity. However, both CR and CURAL groups took fewer trials than AL to reach criterion during the reversal sessions of the active avoidance task, suggesting an improvement in cognitive flexibility. The AL mice had higher levels of CRP compared to CURAL and CR, and GSH as well as the GSH: GSSG ratio were increased during curcumin intake, suggesting a reducing shift in the redox state. The results suggest that, independent of their effects on adiposity; dietary curcumin and caloric restriction have positive effects on frontal cortical functions that could be linked to anti-inflammatory or antioxidant actions.

## Introduction

Complementary and alternative therapies have significant potential to expand current treatment options for obese patients, especially those who can be classified as overweight and class 1 obese yet do not meet clinical criteria for appetite suppressant drugs or bariatric surgery [1]. Very often these classifications can be linked to a sedentary lifestyle, wherein energy intake significantly exceeds that expended, resulting in a chronic positive energy imbalance, weight gain and adiposity. Indeed, a recent report by Sturm and Hattori [2] revealed that only 6.6% of the obese population in the US are morbidly obese, making a strong case for more studies being conducted on alternate therapies for the bulk of the obese population. The current study focused on the therapeutic potential of the polyphenol curcumin, which has gained an increased interest in recent years as a potential treatment for obesity-related comorbidities as well as neurodegenerative disorders [3–5]. Because it has been shown to have weight reducing effects [6–8], administration of curcumin could potentially decrease adiposity and its associated inflammation and oxidative stress.

In testing curcumin as an alternative approach for decreasing adiposity in class 1 obese individuals, the accumulated weight and adiposity of middle-aged, ad libitum (AL)-fed laboratory mice was targeted as a preclinical model. Traditional experimental obesity models (also previously used to study curcumin) have targeted excessive weight gain associated with high fat diets or leptin deficiency [7]. However, the accumulation of weight in these experimental models could be considered a translational analogue of morbid obesity, as these mice often weigh more than two times that of their age-matched controls [7,9]. Moreover, control groups in these studies are fed ad libitum; a condition that itself leads to significant weight gain and adiposity under typical laboratory housing conditions. Recent reviews have highlighted the observation that control rodents kept on ad libitum (AL) feeding often double their weight during adulthood and show an array of obesity-associated conditions by midlife [10,11]. A recent analysis of data on 7 strains of commonly used laboratory rodents suggested that beneficial effects of chronic caloric restriction on life-span were directly proportional to the degree of weight gained by midlife under the AL feeding regimen [12]. These results challenge the use of AL feeding as a control condition and, perhaps more importantly, suggest that under standard laboratory conditions, middle-aged AL fed mice are translational analogues of overweight and mildly obese humans [10]. The Centers for Disease Control and Prevention reports that almost 40% of the US obese population fall within the range of middle age, suggesting that this age group is a significant target for obesity intervention [13].

In addition to addressing therapeutic potential as an anti-obesity medication, it was of interest to determine if curcumin would also influence co-morbid conditions linked to obesity via inflammation and oxidative stress. Mild cognitive impairment has been detected in association with obesity in human studies [14–16], and animal studies of CR imply that improved cognitive function is present in subjects that maintain lower weight [17,18]. Moreover, based on the results from a study conducted on more than 8000 Swedish twins, it was concluded that being overweight or obese during midlife, independent of diabetes and vascular diseases, significantly increased the risk of late life dementia, Alzheimer’s disease and vascular dementia [19]. The same trend has been also reported in several other longitudinal studies with a more diverse population [14–16,20].

Increased levels of inflammatory and oxidative stress markers related to obesity have been linked to impaired cognitive performance in middle-aged individuals in previous studies. For example, inflammatory markers such as interleukin 6 (IL-6) and C-reactive protein (CRP) are reported to be inversely related to cognitive capacity in middle aged obese women [21–23]. Other pro-inflammatory markers such as IL-1β and TNF-α have also been associated with poor memory [24,25]. The association of inflammation with poor cognition may also be influenced by the reciprocal relationship inflammation shares with oxidative stress in several pathologies, which also includes chronic obesity [26–28]. Both IL-6 and TNF-α, whose levels are increased in several pathologies, have been reported to promote the production of reactive oxygen species, a process which reinforces activation of pro-inflammatory transcription factors. A very sensitive indicator of oxidative stress in tissues and blood is the redox state of the endogenous antioxidant glutathione or reduced glutathione (GSH) [29]. Clinically, a decrease in the ratio of reduced to oxidized glutathione in red blood cells has been linked to both mild cognitive impairment and Alzheimer’s disease [29,30]. Further, a decrease in this ratio has also been associated with poor cognitive performance and overall functional decline in several *in vivo* age-associated disease studies [31,32].

The rationale for the current study was to evaluate curcumin as a safe, inexpensive and potentially effective intervention for weight loss, using a preclinical model of midlife sedentary obesity, inflammation, and comorbid cognitive impairment. The effect of curcumin was compared with outcomes for the middle-aged mice subjected to caloric restriction implemented gradually to maintain a healthy weight loss, improve cognition and reduce inflammation. The hypothesis for this study was that dietary curcumin would promote weight loss and reduce adiposity, thereby improving cognition via a concurrent decrease in inflammation and oxidative stress.

## Materials and Methods

### Mice and diets

Fifteen-month-old C57BL/6JNia male mice, maintained at the National Institute on Aging (NIA) colony under ad libitum (AL) feeding of NIH-31, were shipped to UNT Health Science Center where they were assigned in groups of 19 to receive the following dietary regimens for 12 weeks: (i) a base diet fed ad libitum (AL), (ii) the base diet restricted to 70% of AL (CR) or (iii) the base diet containing curcumin fed AL (1000 mg/kg diet, CURAL). Caloric restriction was implemented incrementally (relative to that consumed by AL groups), by 10% of AL during the first week, 20% in the second, and by 30% in the third week. Feed of the CR group remained fixed at this level for the remainder of the experiment. The obese control group (AL) was fed a purified maintenance diet (AIN 93M) (58M1 Test Diet, Richmond, IN) (S1 Table) ad libitum. The CURAL group was fed the same maintenance diet supplemented with 1000 mg of curcumin (Sigma-Aldrich, St. Louis, MO)/kg of diet (Catalog no: 1815457–201) (S1 Table). The CR group received a diet fortified with vitamins and minerals formulated by Test Diet (Catalog no: 1815458–209) (S1 Table). All animals were fed the AL diet for 2 weeks to acclimate them on the purified diet (because they were fed a grain-based diet at NIA), following which they were randomly assigned to one of the three treatment groups. Body weight was measured once every week, and food intake for CURAL and AL was estimated at 4, 8 and 12 weeks of treatment. Behavior tests were initiated after 8 weeks of dietary treatment and continued until the last week of the treatment period. Mice underwent tail bleeds at 8 and 12 weeks following treatment, and visceral and subcutaneous adipose tissue (which include epididymal and gluteal adipose tissue respectively) was collected after mice had been euthanized at the conclusion of the study. This study was carried out in strict accordance with the recommendations in the Guide for the Care and Use of Laboratory Animals of the National Institutes of Health. The animal protocol was approved by the University of North Texas Health Science Center Institutional Animal Care and Use Committee in Fort Worth, TX (Protocol no: **2011/12-30-A04**).

### Assessment of systemic inflammation

All protein concentrations were determined using the BCA Protein Assay Kit from Pierce Biotechnology (Rockford, IL) with bovine serum albumin as a standard. Serum was separated from blood and the inflammatory markers were measured using ELISA. CRP was measured using a mouse CRP kit from R&D Systems (Minneapolis, MN) and IL-6 was measured using a kit from Life Technologies (Carlsbad, CA).

### Assessment of redox state

Prior to the processing of erythrocytes, the collected blood was centrifuged at 2,500g for 10 min at 4°C; the plasma was removed to be analyzed for inflammatory cytokines. The white blood cells were removed and the remaining red blood cells were lysed with 4 times its volume of ice cold double distilled water and incubated for 10 min on ice. The samples were then centrifuged at 10,000g for 15 min at 4°C. Some of the supernatant was collected to measure protein concentration and the rest received an equal volume of metaphosphoric acid to stop the oxidation process and stored at -80°C until assayed. Total glutathione (tGSH) and oxidized glutathione (GSSG) were measured from erythrocytes collected during the blood draws according to manufacturer’s instructions, and the concentration of reduced glutathione (GSH) was then calculated using the formula: tGSH-(2*GSSG) /GSSG and GSH: GSSG was calculated. The GSH: GSSG kit was purchased from Cayman Chemical (Ann Harbor, MI).

### Cognitive skills assessment

At the end of week 8 of dietary treatment, mice underwent behavior tests to analyze domains of cognitive performance linked to hippocampal (spatial learning) versus frontal cortical (cognitive flexibility) functions.

### Spatial learning and memory

Spatial learning and memory were measured using a Morris water maze (MWM) test similar to that described previously [33–35]. This test provided a measure of the efficiency of mice in swimming to a hidden, safe platform from different locations within a cylindrical tank filled with opacified water. A pre-training phase was conducted in two sessions during which mice did not have access to spatial information (cues) in the testing room, to ensure that the mice had learned the motor components of swimming and climbing onto the platform prior to testing for spatial performance. During subsequent testing, a computerized tracking system recorded the length of the path taken by the mouse to reach the platform, as well as the swimming speed (Any-maze; Stoelting Co., Wood Dale, IL, USA), with spatial cues available in the open tank and the hidden platform in a fixed location. Testing was conducted over nine sessions (1–4, Tuesday-Friday and 5–9, Monday-Friday the following week). Each session consisted of five trials separated by an ITI of 90s during which the mouse had to swim to the platform from one of four different starting points. A learning index was calculated as the average path length on sessions 2, 3 and 4, the initial learning phase. Probe trials were conducted immediately following sessions 2, 4, 5, 7, and 9, during which the platform was submerged to a depth that prevented the mice from climbing onto it. The platform was raised after 30 s, and the trial was ended when the mouse successfully located it. The percentage of time spent in a 40 cm diameter annulus around the platform location was calculated as a measure of spatial bias for the platform location. Memory retention for the platform location was tested during one additional session conducted one week after session 9.

### Cognitive flexibility

An acrylic T-maze (black on the sides with a clear top) was used for the discriminated avoidance task [33,36,37]. The maze was divided into three compartments with a stem and goal arms that rested on a grid floor wired to deliver 0.67-mA scrambled shock to the feet of the mice. The test consisted of three sessions separated by 1 h. Each mouse was trained to leave the start box and run to a designated correct goal within 5 s after lifting of the start door. On the first trial of the first session (acquisition), the correct goal arm was designated as the one opposite from the mouse’s first arm choice (determined on a separate preference trial). On subsequent trials, shock was initiated 5 s after the opening of the start door if the mouse had not entered the correct goal arm, or immediately upon entry into the incorrect arm. In both cases, shock was continued until the correct goal arm was entered or a maximum of 60 s had elapsed. A correct avoidance trial was recorded when the mouse entered the correct goal arm within 5s of the opening of the start door. The mouse was then allowed to stay in the correct goal arm for 10 s after which the mouse was removed and placed in a holding cage for 1 min. A session ended when the mouse had made a correct avoidance on the last two trials and on at least 4 of the last 5 trials. The second and third sessions of avoidance training were reversals such that the mice were required to run to the goal arm opposite that to which they had previously been trained. Ability to learn the avoidance problem was considered inversely proportional to the number of trials required to reach criterion in each of the sessions. Learning efficiency during the second and third sessions was considered indicative of cognitive flexibility [33,37].

### Statistical Analysis

Measures of performance on the MWM test were initially considered in a 3 x 2 x 4 analysis of variance (ANOVA) with Diet as a between-groups factor and Test phase (session 1–4 vs 5–8) and Session as within-subject factors. The number of trials required to reach the correct avoidance criterion was considered in a two-way ANOVA with Diet as a between groups factor and Sessions as a within subject factor. In the context of a significant main effect of Diet or a Diet x Session interaction, planned comparisons were conducted between diet groups using single degree of freedom F tests within the Diet x Session interaction (i.e., using the cell means and overall error term). A similar approach was applied to analysis of data for body weight and food intake, with weeks as the within subject factor. One-way between groups ANOVA was applied to data for CRP, IL6, GSH, GSH: GSSG and GSSG, followed by planned comparisons among diet groups. The alpha level was set to 0.05 for all analyses.

## Results

**Fig 1:**
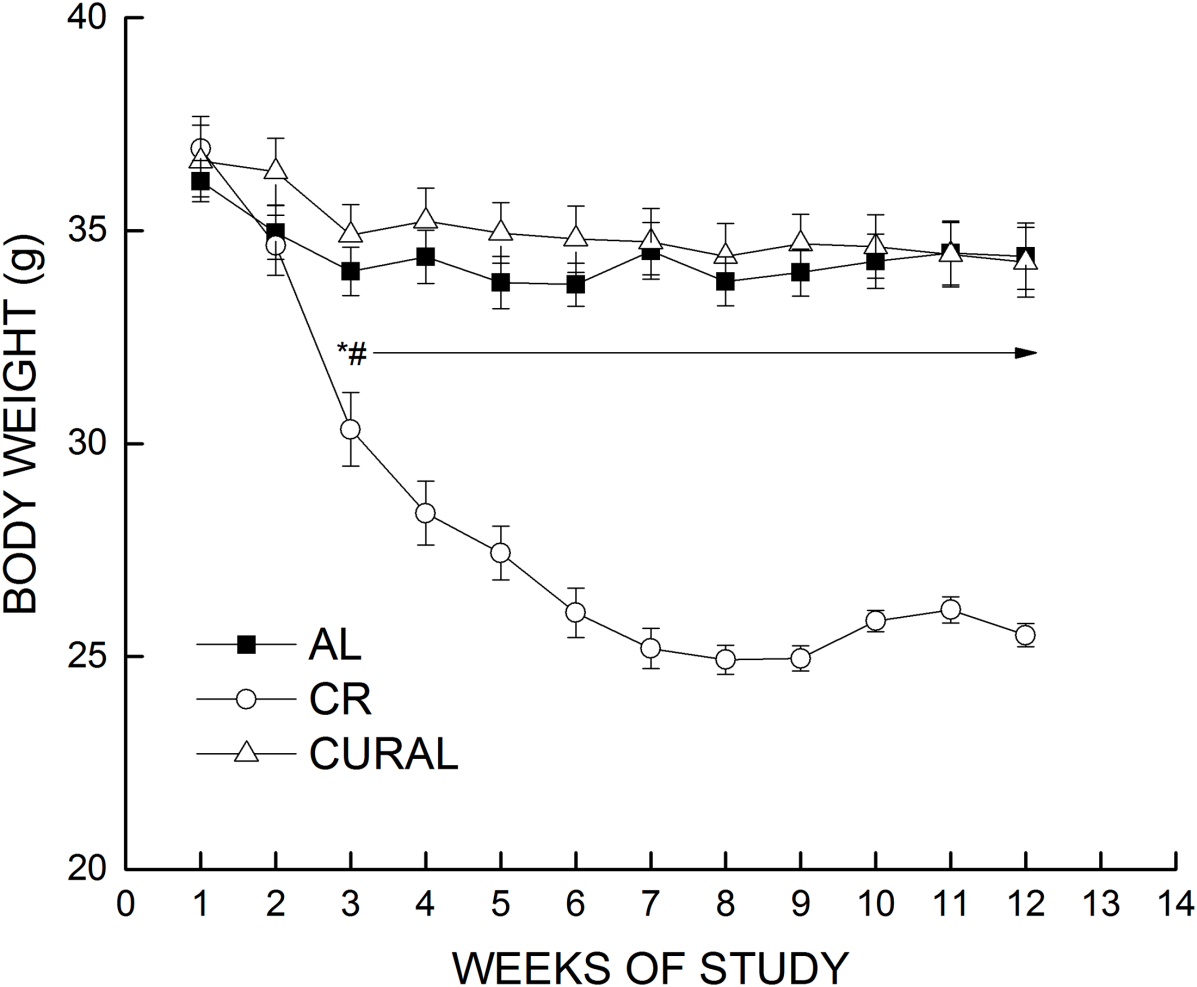
Body weight over a span of 12 weeks of dietary treatment. Data are means ±SEM, n = 18–19, for: AL, base diet fed ad libitum; CR, base diet restricted to 70% AL, CURAL, curcumin in base diet fed AL. Symbols indicate differences, *P* < 0.05: (a) different from AL (*), (b) different from CURAL (#).

**Fig 2:**
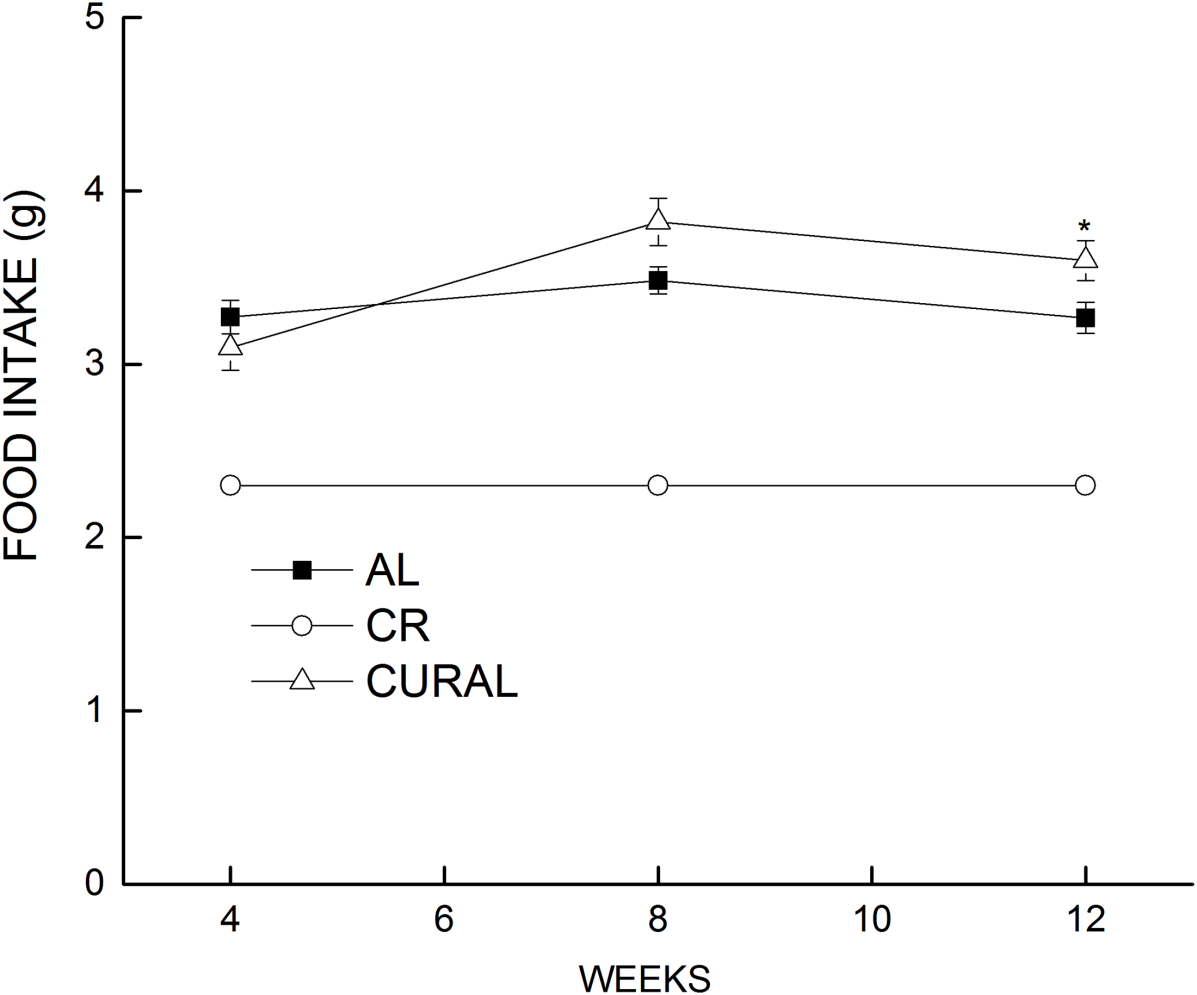
Estimated food intake over a span of 12 weeks of dietary treatment. Data are means ±SEM, n = 18–19. Food intake was measured at 4, 8 and 12 weeks of dietary treatment. Symbols indicate differences, *P* < 0.05: (a) different from AL (*).

Body weight, food intake and adiposity. The CR group had significant loss of body weight compared to mice on curcumin or AL beginning in week 3, and they continued to lose weight until week 6, after which they maintained stable weight for the rest of the treatment period (Fig 1). Both CURAL and AL groups maintained stable body weight throughout the 12-week dietary treatment, and there was no difference in body weight between the CURAL and AL group at any time point. However, in spite of the lack of difference in body weight compared to AL mice, CURAL mice had a higher food intake (almost 25%) compared to AL at week 12 of the dietary treatment period (Fig 2). Analyses of variance for body weight and food intake supported these observations, revealing a significant effect of diet, weeks, and the interaction of those factors (All *P*<0.03).

**Fig 3:**
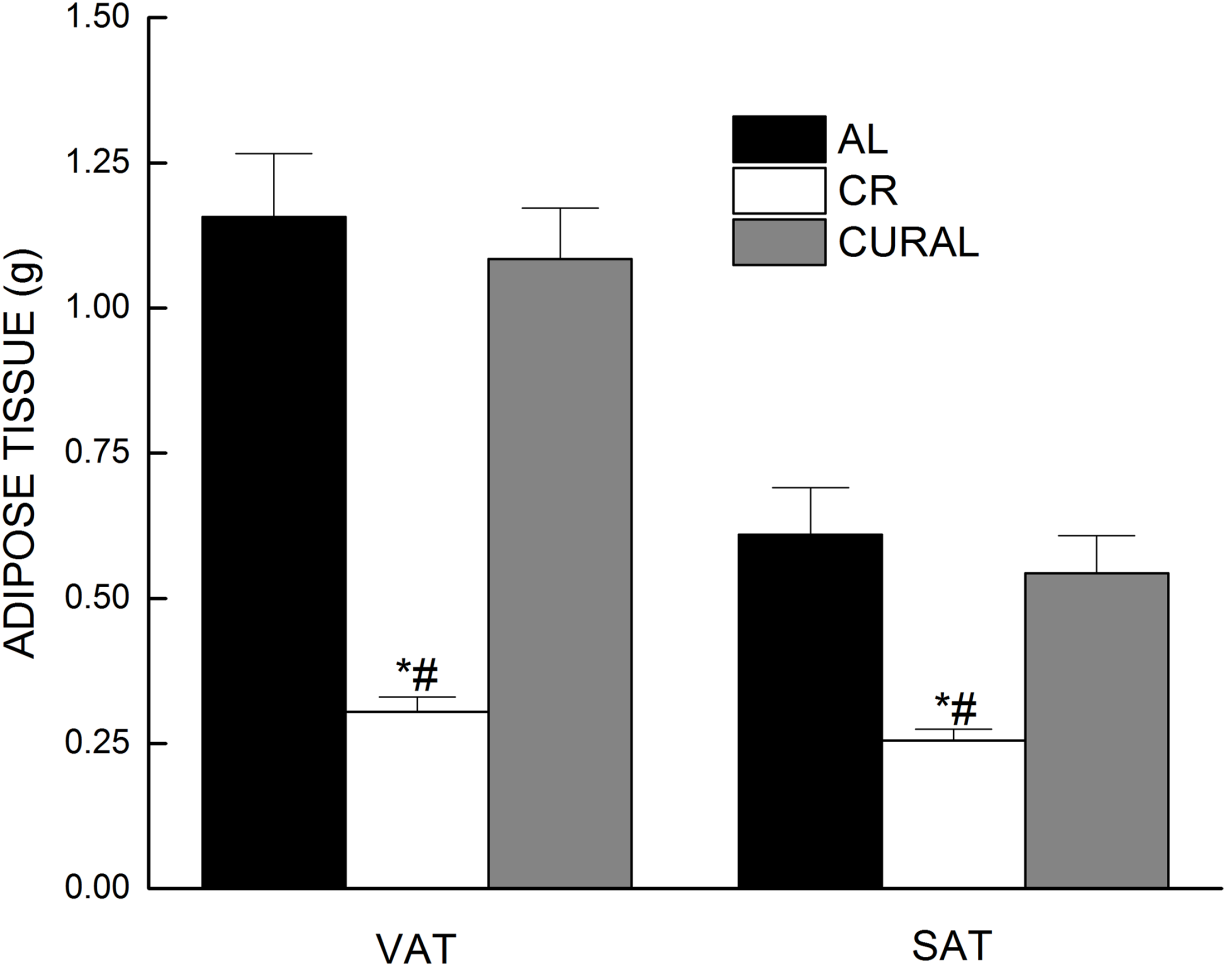
Adipose tissue weights after 12 weeks of dietary treatment. Data are means ± SE, n = 18–19. Visceral adipose tissue (VAT) was collected from epididymis and, subcutaneous adipose tissue SAT was collected from the gluteal region. Symbols indicate differences, *P* < 0.05: (a) different from AL (*), (b) different from CURAL (#).

At the end of the 12-week treatment, the CR group had markedly lower amounts of both VAT and SAT when compared with the AL group (Fig 3), but there was no decrease in adipose tissue associated with intake of curcumin. The fat loss in the CR group was responsible for a main effect of diet in analyses of variance on visceral and subcutaneous adipose tissue (all *P*<0.001).

### Inflammation and redox state

**Fig 4:**
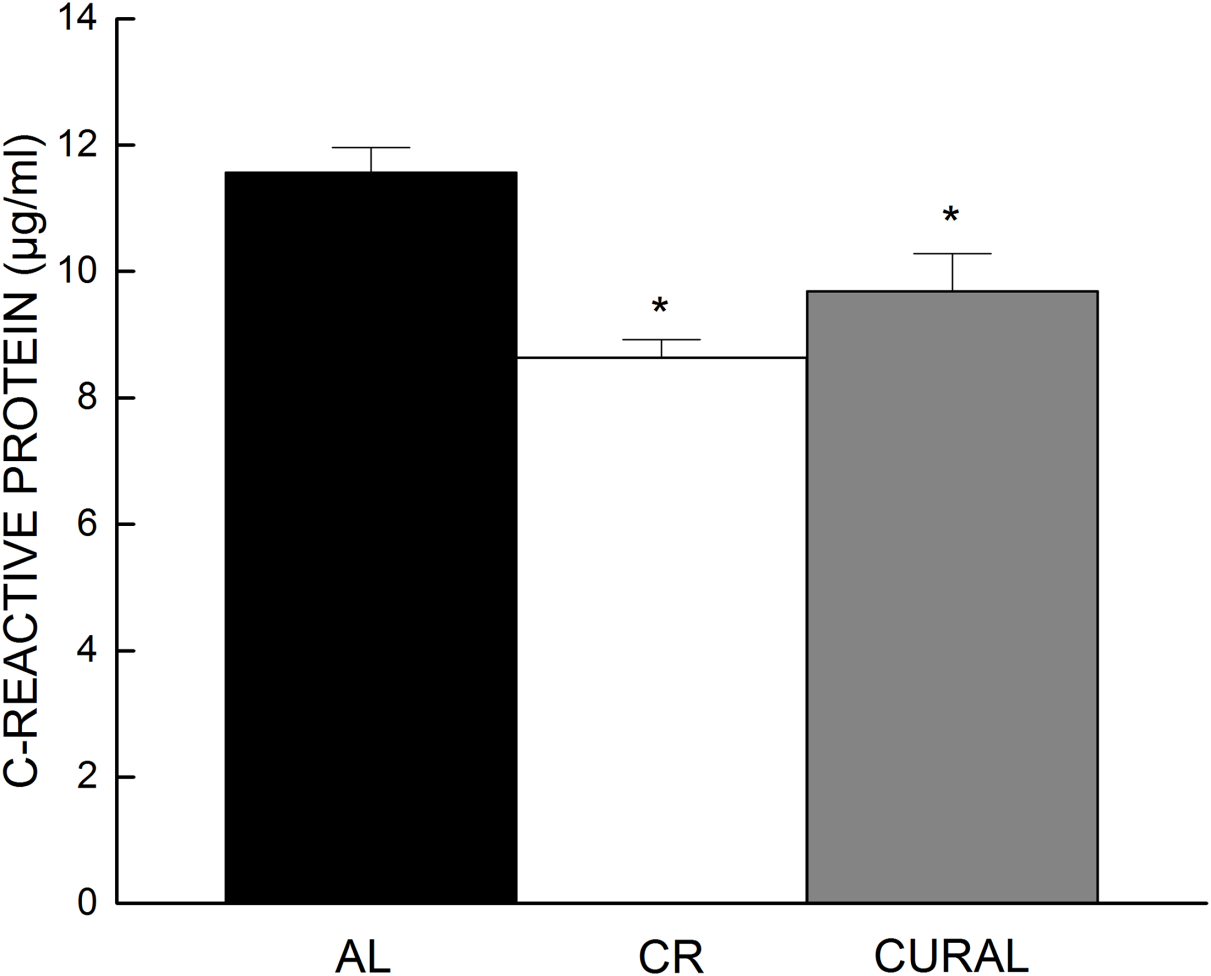
Serum concentration of C-reactive protein as a function of diet. Data represent the average of measurements at 8 and 12 weeks of treatment. Bars represent means +SE, n = 8–10. (a) *P*<0.05 when compared with AL (*).

**Fig 5:**
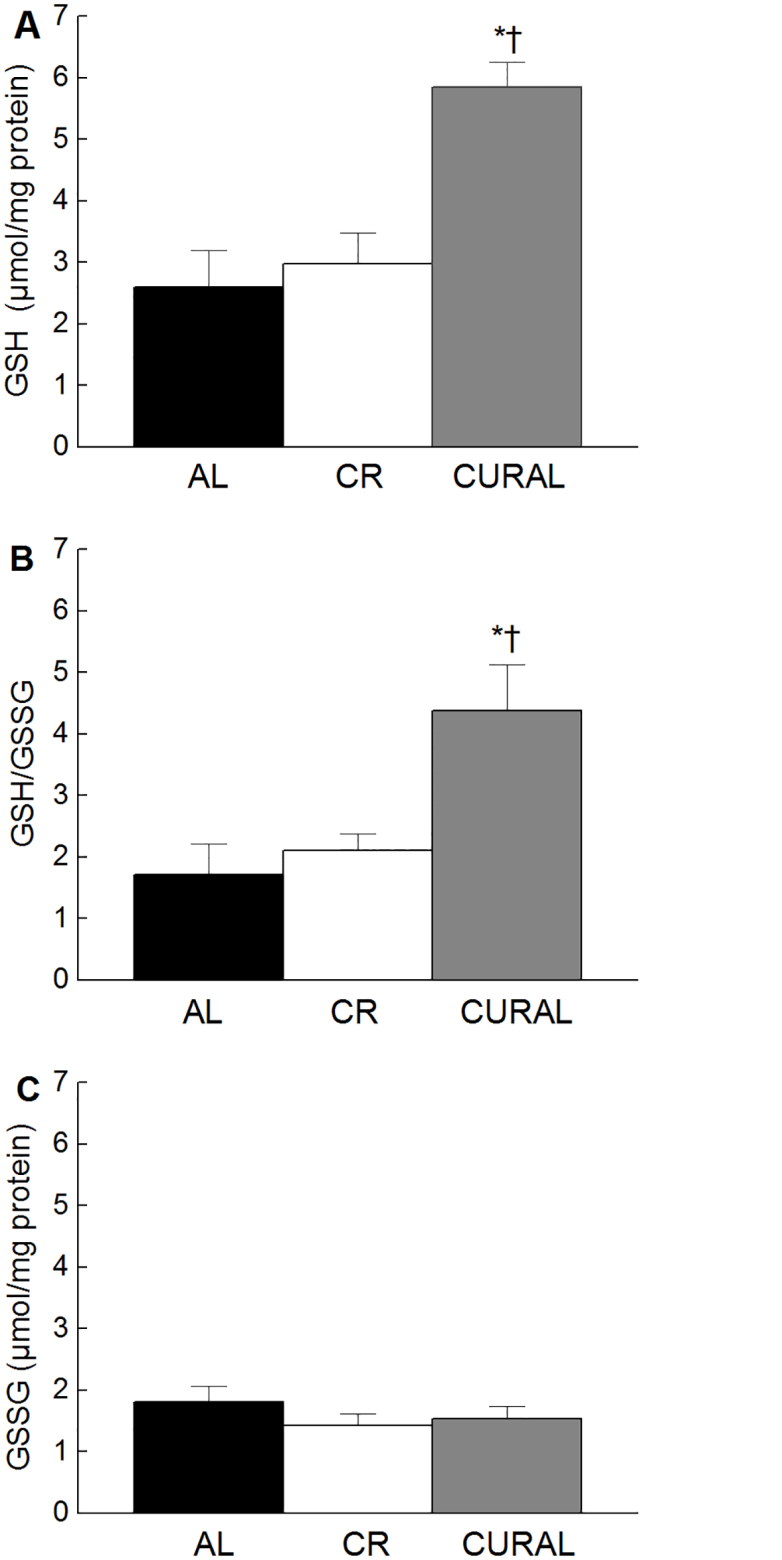
Redox state. Concentration of (A) reduced glutathione, (B) GSH/GSSG and (C) oxidized glutathione in erythrocytes after 8 weeks of dietary treatment. Bars represent means ± SE, n = 8–10. Symbols indicate differences, *P* < 0.05: (a) different from AL (*), (c) different from CR (†).

There was no significant effect of diet on concentration of IL-6 at 8 or 12 weeks (all *P*<0.27, S2 Table). However, the obese control (AL) on average had a higher concentration of serum CRP than the CURAL or CR group at both 8 and 12 weeks of treatment. Data analysis indicated a significant main effect of Diet (*P* = 0.006) in the absence of an interaction with weeks (*P* = 0.770) (Fig 4). Curcumin-treated mice had a higher concentration of reduced glutathione (Fig 5a) and a more reducing redox state (GSH: GSSG) compared to mice on CR and those fed AL (all *P* < 0.05) (Fig 5b). There was, however, no difference in GSSG among the different diet groups (*P* >0.446) (Fig 5c).

### Spatial learning and cognitive flexibility

**Fig 6:**
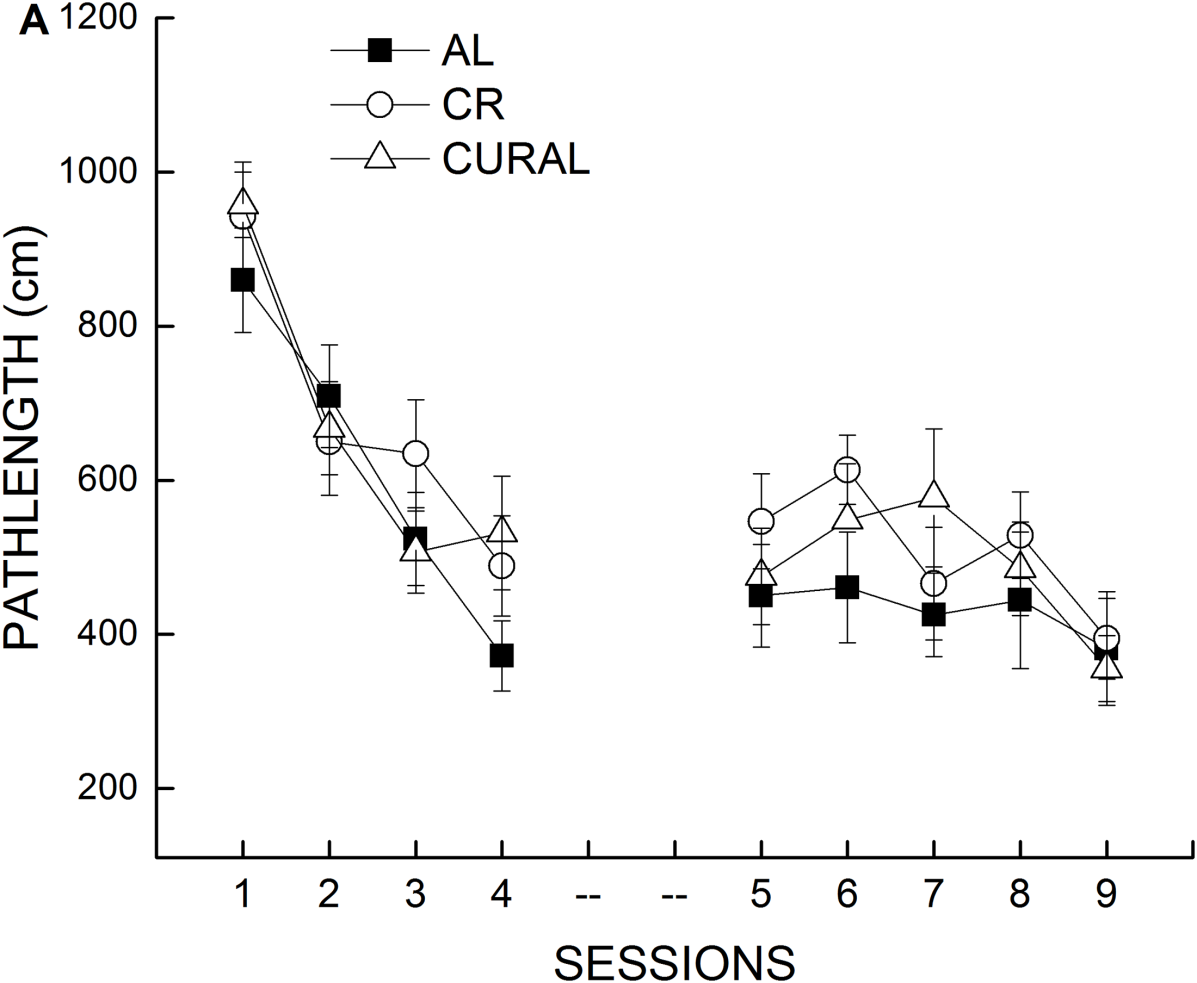
Water maze performance. The effect of dietary treatment on the efficiency to locate a hidden platform (path length) as a function of training sessions. All data represent means ± SE, n = 18–19.

The distance to reach the hidden platform (path length) was determined during sessions 1–9 to assess the efficiency with which the mice located the platform regardless of their swimming speed. Over sessions 1–4, all groups showed improvement in their efficiency to reach the platform (Fig 6), and there was no apparent difference among the treatment groups in the spatial learning index (S2 Table) or during probe trials conducted after sessions 2 and 4 (data not shown). Performance during the second week remained relatively stable for the treatment groups. Data analysis failed to indicate a significant effect of diet or interaction of diet with test phase or session (all *P*>0.62). Probe trial data calculated from percent time spent in the 40-cm annulus around the target site (not shown) confirmed that mice in the different treatment groups had acquired a spatial bias for the platform location that was maintained through session 9. However, analysis of the probe data failed to indicate a significant main effect or interaction involving diet (all *P*>0.15).

**Fig 7:**
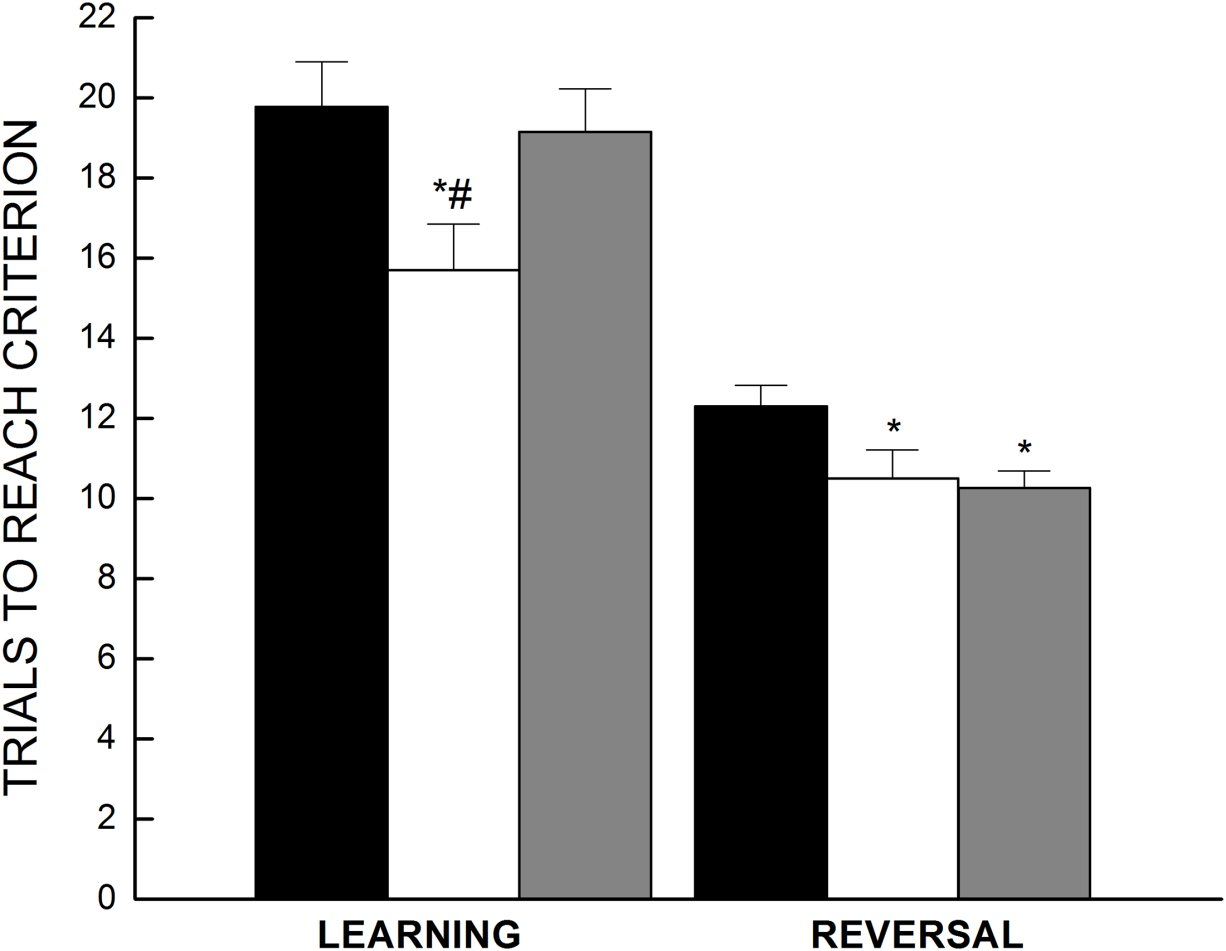
Active avoidance performance. Effect of dietary treatment on learning and reversal of an active avoidance response to one of two locations in a T-maze. During the first session mice learned the avoidance response, and reversals of the correct location occurred during two subsequent sessions which were combined (AVG) to assess cognitive flexibility. All data are expressed as the mean number of trials (± SE) needed to meet the correct avoidance criterion, n = 18–19. Symbols indicate differences, *P* < 0.03: (a) different from AL (*), (b) different from CURAL (#).

Cognitive flexibility was tested by first establishing a discriminated avoidance response, followed by two additional sessions separated by 1 hour in which the required correct response was reversed. Fig 7 displays the initial learning versus the average number of trials taken during sessions 2 and 3 to reach criterion (reversal). Mice kept on CR took fewer trials to reach criterion compared to AL and CURAL in the initial learning phase. During the reversal sessions, both the CR and CURAL groups took fewer trials to reach criterion when compared with AL. A two-way ANOVA on the acquisition phase and reversal phase with diet and phase as the independent variables confirmed the mentioned observations, all *P*<0.03 (Fig 6).

## Discussion

The effects of curcumin have not previously been considered in a model of midlife sedentary obesity. The main findings of this study were as follows: 1. Curcumin was ineffective in decreasing adiposity, but increased apparent energy intake in the absence of weight gain; 2. Calorically restricted mice lost both weight and had significantly reduced visceral and subcutaneous adipose tissue; 3. Curcumin intake promoted a more reducing redox state and a lower level of inflammation and 4. Curcumin and caloric restriction improved executive function but did not affect spatial learning. Overall, these results suggest that dietary curcumin produces an improvement of cognitive function similar to CR, in the absence of a significant effect on body weight or adiposity. This effect may involve an anti-inflammatory effect similar to that of CR, or an antioxidant mechanism.

Caloric restriction has been extensively studied in mice and other non-human primates. These studies have suggested that CR can extend lifespan and delay onset of age-related diseases for which oxidative stress and inflammation are also established risk factors [12,38–40]. While it has been argued extensively that CR attenuates aging and disease processes independently of the effect on adiposity, it remains likely that the anti-obesity effect is a key factor [38,41]. In this study, a 12-week regimen of CR, implemented gradually, not only reduced adiposity, but also improved frontal cortical functions and decreased plasma C-reactive protein (CRP), a biomarker of systemic inflammation. The beneficial effect of long-term CR on discriminated avoidance learning has been reported previously [17,18], although the current findings suggest additionally that this outcome is evident after only 3 months of CR, when its implementation is delayed until middle age. This outcome favors the view that apparent long-term benefits of CR regimens on cognition are at least partly attributable to reversible physiological actions [42–44]. However, in apparent contrast to previous investigations, there was no effect of short-term CR on oxidative stress, when measured as the ratio of GSH: GSSG in erythrocytes [39]. The different outcome in the current and previous studies of CR could be attributed to a difference in severity of the CR regimens (30 vs. 40%), the approach to measurement of GSH and GSSG, or the duration of the regimens.

Although the beneficial effects of CR are well known, it has been difficult to translate this regimen to a clinical setting because of the reluctance of most individuals to change eating behavior and modify food choices [45]. A sustainable alternative or adjunct to the practice of caloric restriction may involve compounds that mimic the beneficial effects of caloric restriction and do not require a change in eating habits [46]. Curcumin, a widely studied polyphenol, has gained significant interest as a caloric restriction mimetic, and our current findings of improved cognitive function and attenuated inflammation and oxidative stress would seem to confirm this suggestion. Moreover, despite the absence of weight loss in the CURAL group, the current studies do not rule out the possibility that curcumin intake is associated with a metabolic effect. The mice under the curcumin regimen showed a noticeable increase in food intake relative to their age-matched obese control, yet no net change in energy balance was detectable as weight gain. This finding would imply that curcumin increases energy expenditure, although no measurements of respiration, body temperature or physical activity were available from these studies for confirmation of this hypothesis. Several papers have reported curcumin’s ability to activate AMP kinase (AMPK) a cellular energy sensor and stimulant of ATP production implicated as a physiological mechanism of the CR effect [7,47,48]. Kim et al [49] found that both curcumin and its active metabolite tetrahydrocurcumin were effective in activating AMPK and inactivating Acetyl CoA carboxylase. Such an increase in energy expenditure would be offset by an increase in energy intake under conditions of AL feeding.

The curcumin-fed mice also had low levels of systemic inflammation, which was measured via serum CRP, widely considered a translational index of low grade inflammation [50,51]. Mice under caloric restriction also had lower levels of CRP compared to the obese control (AL). These findings are consistent with previous reports that both curcumin and caloric restriction have strong anti-inflammatory effects [6,41,52–55]. For mice under CR, it seems likely that the attenuation of inflammation can be directly linked to reduction of adiposity. However, the lack of effect of curcumin on adiposity suggests a different anti-inflammatory mechanism of action. Previous studies have reported on curcumin’s ability to decrease inflammation via the inhibition of the nuclear translocation of nuclear factor kappa-light-chain-enhancer (NF-κB) of activated B cells. The nuclear translocation of NF-κB results in the transcription of pro-inflammatory cytokines such as IL-6, IL-1β and TGF-β. These cytokines can then induce CRP synthesis by hepatocytes [56]. In vitro studies have reported curcumin binding to IKKβ and thereby preventing the activation of this kinase leading to downstream inhibition of NF-κB activity [57].

Despite the effects of curcumin and CR on CRP, no effect of treatments on serum IL-6 was evident in the current study. However, TNF- α is a stronger inducer of CRP than IL-6 and IL-1β alone [58], and is expressed in and secreted by adipocytes. The concentration of TNF- α correlates with the degree of visceral adiposity and, based on the current study, CR significantly reduced adipose tissue whereas curcumin did not. For this reason, curcumin and CR may have acted via different pathways to reduce inflammation. Additionally, both CR and curcumin were hypothesized to improve redox state; however, only dietary curcumin improved the redox state. There was a significant increase in both reduced glutathione and total glutathione but no significant differences in oxidized glutathione levels amongst the groups, suggesting that curcumin may increase glutathione synthesis. Curcumin has previously been reported to up-regulate the expression of Nuclear factor (erythroid-derived 2)-like 2 (Nrf2) via conformational change of Kelch ECH associating protein 1 by either alkylation or oxidation, thereby allowing the nuclear translocation of Nrf2 and increasing glutathione synthesis [59,60]. Although CR has been reported to do the same [61], the lack of an increase in reduced glutathione could be associated with the shorter intervention period implemented in this study. In addition, several studies have reported on curcumin’s strong anti-oxidant activity, which can be attributed to its molecular structure enabling it to be a strong free radical quencher [54,62,63].

Previous studies from our laboratory as well as others have determined impaired cognition to be strongly associated to increased oxidative stress. Indeed, lower reduced to oxidized glutathione ratios are correlated with poorer cognitive performance [29,30,32]. Our findings further strengthen this suggestion, with mice under the curcumin regimen displaying better executive function than the obese controls. However, the same cannot be concluded with regard to mice under the caloric restriction regimen. Previous studies have also reported on a strong association of inflammation with cognition, which may be the contributing factor for better executive function and acquisition that occurred in mice under the CR regimen in the current study. Although both of these treatments had positive effects for fronto-cortical functioning, they did not have any effect on hippocampal dependent spatial learning. Neither the learning nor overall performance on the spatial learning task was affected by the interventions. This finding is congruent with the progression of cognitive dysfunction as evident from neurological assessments in the non-demented aging population. More often than not, older dementia free individuals suffer from difficulties on tasks that stress attention, cognitive flexibility and other executive functions [64–66] than hippocampal-dependent spatial learning and long-term memory. Results from the current study and that from human cognitive evaluations may indicate curcumin to be a potential therapeutic for fronto-cortical dysfunction reported with normal aging.

Finally, the dosage we used for this study was decided based on a previous obesity related study and translates roughly to a human dose of only 500mg [7]. Future studies should test a higher translatable dose corresponding to suggested doses for individuals suffering from severe inflammation-related diseases such as rheumatoid arthritis. Based on the calculation that uses the body surface area and metabolic activity of the species index by Reagan-Shaw et al. the human equivalent dose in this study falls below the 1500mg that has been recommended for daily use [67]. Follow-up studies with higher doses may further elucidate on a dose-response related to cognitive function.

In conclusion, the present findings from this study did not support the hypothesis that reduction of adiposity is responsible for improved cognition following curcumin intake. However, our findings suggest that both caloric restriction and dietary curcumin can improve obesity-associated comorbidities.

## Supporting Information

S1 Table Composition of control (AL), CR and curcumin (CURAL) diets. ^1.^ All diets were formulated and prepared at Test Diet, Richmond, IN. ^2.^ Curcumin (C1386) was purchased in powder form from Sigma-Aldrich, St. Louis, MO and sent to Test Diets to be added to CURAL diet.
(DOCX)

S2 Table Effect of diet on adipose tissue, inflammation, oxidative stress and memory. ^**1.**^ *P* <0.05 compared with AL.
(DOCX)
