# Supplementary material for: Curcumin Mimics the Neurocognitive and Anti-Inflammatory Effects of Caloric Restriction in a Mouse Model of Midlife Obesity
Source: PLoS One. 2015 Oct 16;10(10):e0140431. doi: 10.1371/journal.pone.0140431 (PMC4608712; doi:10.1371/journal.pone.0140431)
Supplement: S1 Table — 1. All diets were formulated and prepared at Test Diet, Richmond, IN. 2. Curcumin (C1386) was purchased in powder form from Sigma-Aldrich, St. Louis, MO and sent to Test Diets to be added to CURAL diet. (DOCX) [file pone.0140431.s001.docx]

**ONLINE SUPPLEMENTARY MATERIAL**

| **Ingredient** (g/kg diet) | **AL^1^** | **CR^1^** | **CURAL^1,2^** |
| --- | --- | --- | --- |
| Casein | 140.0 | 140.0 | 140.0 |
| Curcumin | 0 | 0 | 1.0 |
| L-Cystine | 1.8 | 1.8 | 1.8 |
| Sucrose | 100.0 | 100.0 | 100.0 |
| AIN 93M Mineral mix | 35.0 | 35.0 | 35.0 |
| AIN 93M Vitamin mix | 10.0 | 20.0 | 10.0 |
| Corn starch | 448.0 | 455.0 | 458.0 |
| Cellulose | 50.0 | 50.0 | 50.0 |
| Choline bitartarate | 2.5 | 2.5 | 2.5 |
| Soybean oil | 40.0 | 40.0 | 40.0 |
| Maltodextrin | 155.0 | 155.0 | 155.0 |

1. All diets were formulated and prepared at Test Diet, Richmond, IN
2. Curcumin (C1386) was purchased in powder form from Sigma-Aldrich, St. Louis, MO and sent to Test Diets to be added to CURAL diet

Supplemental Table 1: Composition of control (AL), CR and curcumin (CURAL) diets.
