# Supplementary material for: Curcumin Mimics the Neurocognitive and Anti-Inflammatory Effects of Caloric Restriction in a Mouse Model of Midlife Obesity
Source: PLoS One. 2015 Oct 16;10(10):e0140431. doi: 10.1371/journal.pone.0140431 (PMC4608712; doi:10.1371/journal.pone.0140431)
Supplement: S2 Table — 1. P <0.05 compared with AL. (DOCX) [file pone.0140431.s002.docx]

**ONLINE SUPPLEMENTARY MATERIAL**

|  | **AL** | **CR** | **CURAL** |
| --- | --- | --- | --- |
| **Adipose tissue** |  |  |  |
| Visceral (g) | 1.16±0.11 | 0.31±0.03**^1^** | 1.01±0.09 |
| Subcutaneous (g) | 0.61±0.08 | 0.26±0.02**^1^** | 0.54±0.07 |
|  |  |  |  |
| **Biochemical markers** |  |  |  |
| IL-6 (pg/ml) | 13.0±3.38 | 31.2±7.21 | 21.0±5.73 |
| CRP (μg/ml) | 11.9±0.86 | 10.0±0.73^1^ | 9.18±0.44^1^ |
| GSH (μmol/mg protein) | 2.59±0.60 | 2.97±0.50 | 5.85±0.41^1^ |
| GSSG (μmol/mg protein) | 1.81±0.25 | 1.43±0.18 | 1.53±0.21 |
| GSH:GSSG | 1.72±0.50 | 2.11±0.30 | 4.38±0.75^1^ |
|  |  |  |  |
| **Memory** |  |  |  |
| Learning Index (cm) | 549±32.7 | 591±42.2 | 569±41.7 |
| Acquisition (trials) | 19.8±1.13 | 15.7±1.14^1^ | 19.2±1.07 |
| Reversal (trials) | 12.3±0.53 | 10.5±0.71^1^ | 10.3±0.43^1^ |

**^1.^** *P* <0.05 compared with AL

Supplemental Table 2: Effect of diet on adipose tissue, inflammation, oxidative stress and memory
